# Patient-derived resources for decoding and targeting brain metastases ecosystems

**DOI:** 10.1038/s44321-026-00472-y

**Published:** 2026-07-03

**Authors:** Manuel Valiente, Lal Era Aydoğan, Nisan Feigin, Clara Gahn, Carolina Hernandez-Oliver, Friedrich Milling, Evren Oktem, Carmen Ortega-Sabater, Neibla Priego, Alessia Andrea Ricci, Giulia Capella, Luca Mangherini, Laura Serrano-Ron, Antonia Stammberger, Juan Vazquez-Canto, Katharina J Weber, Rebekka Wehner, Thomas Broggini, Marcus Czabanka, Serap Aksu, Fatima Al-Shahrour, Luca Bertero, Marc Schmitz, Itay Tirosh, Hind Medyouf, RISEBrain Consortium, Manuel Valiente, Lal Era Aydoğan, Nisan Feigin, Clara Gahn, Carolina Hernandez-Oliver, Friedrich Milling, Evren Oktem, Neibla Priego, Alessia Andrea Ricci, Giulia Capella, Luca Mangherini, Laura Serrano-Ron, Antonia Stammberger, Juan Vazquez-Canto, Rebekka Wehner, Serap Aksu, Luca Bertero, Marc Schmitz, Itay Tirosh, Hind Medyouf

**Affiliations:** 1 Brain Metastasis Group, Spanish National Cancer Research Centre, Madrid, Spain; 2 Department of Hematology, Oncology, Hemostaseology, Stem Cell Transplantation, Medical Faculty, Center for Integrated Oncology (CIO) RWTH Aachen University, Aachen, Germany; 3 Department of Molecular Cell Biology, Weizmann Institute of Science, Rehovot, Israel; 4 Institute of Immunology, Faculty of Medicine Carl Gustav Carus, TUD Dresden University of Technology, Dresden, Germany; 5 Biomedical Science and Engineering, Koç University, Istanbul, Türkiye; 6 CNIO Biobank, Spanish National Cancer Research Centre, Madrid, Spain; 7 Pathology Unit, Department of Medical Sciences, University of Turin, Turin, Italy; 8 Bioinformatics Unit, Spanish National Cancer Research Centre (CNIO), Madrid, Spain; 9 Goethe University Frankfurt, Neurological Institute (Edinger Institute), Frankfurt am Main, Germany; 10 Frankfurt Cancer Institute (FCI), Goethe University Frankfurt, Frankfurt am Main, Germany; 11 National Center for Tumor Diseases (NCT/UCC) Dresden, a partnership between DKFZ, Faculty of Medicine and University Hospital Carl Gustav Carus, TUD Dresden University of Technology, and Helmholtz-Zentrum Dresden-Rossendorf (HZDR), Dresden, Germany; 12 German Cancer Consortium (DKTK), Partner Site Dresden, and German Cancer Research Center (DKFZ), Heidelberg, Germany; 13 Department of Neurosurgery, University Hospital Frankfurt, Goethe University Frankfurt, Frankfurt am Main, Germany

**Keywords:** Cancer

## Abstract

Brain metastases (BrM) affect up to 30% of patients with solid tumors, yet durable intracranial control remains rare, and the biological drivers of this poor prognosis are incompletely understood. Patient-derived resources, such as clinical cohorts, biobanks, functional ex vivo models, and multi-omic platforms, are central to closing this gap, but their generation and integration face substantial logistical and technical hurdles. Drawing on the RISEbrain consortium's experience, this Perspective examines BrM-focused cohorts and biobanks, highlighting the underused potential of rapid autopsy programs to capture early metastatic seeding. We discuss patient-derived organotypic cultures and emerging organoid-based "avatar" systems as functional platforms for therapeutic profiling, alongside the complementary strengths of bulk and single-cell/-nucleus transcriptomics. We outline how spatial transcriptomics and proteomics are resolving the architecture of the BrM microenvironment, and assess liquid biopsy approaches, including emerging photonic biosensors, for non-invasive monitoring. Together, these resources form an interdependent toolkit whose coordinated deployment will advance early detection, prevention, and precision treatment of BrM.

## Abstract (web summary)

This Perspective by H. Medyouf, M. Valiente and collaborators highlights the complexity of studying brain metastases, particularly in relation to patient-derived resources, and provides an overview of several ongoing initiatives.

## Graphical abstract

**Figure:**
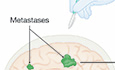

## Introduction

Brain metastases (BrM) are the most common intracranial malignancies, affecting up to 30% of adult cancer patients and causing substantial morbidity and mortality, particularly in lung, breast, melanoma, and colorectal cancers (Valiente et al, 2025). Despite advances in local and systemic therapies, median survival after BrM diagnosis remains limited, and durable intracranial control is achieved in only a subset of patients. These challenges highlight the need for a deeper biological understanding of BrM as organ-specific ecosystems and for translational strategies leveraging patient-derived resources to improve prevention and treatment.

Over the past years, BrM research has shifted from descriptive pathology to integrative molecular and functional analyses. Key advances include recognition of BrM as biologically distinct from primary tumors, identification of brain-specific tumor–microenvironment interactions, and development of technologies such as spatial omics, patient-derived models, and integrative computational approaches (Valiente et al, 2025). Nonetheless, major gaps persist, including limited predictors of brain involvement, incomplete insight into early metastatic seeding, and barriers to clinical translation. This Perspective draws on the RISEbrain consortium to assess progress, remaining limitations, and future directions for targeting BrM ecosystems.

## The use of clinical cohorts for brain metastasis research

### Clinical context and unmet needs

Approximately 40% of patients with BrM undergo neurosurgical resection as part of local treatment, although this proportion varies widely across institutions. Surgical decision-making depends on multiple variables, including patient performance status, extracranial disease burden, number and anatomical location of brain lesions, and anticipated clinical benefit (Soffietti et al, 2020). Importantly, surgery is rarely curative and is primarily performed for symptom control or diagnostic purposes (Soffietti et al, 2020). Nevertheless, surgical access provides a unique and often irreplaceable opportunity to obtain fresh human tissue, which is essential for mechanistic studies and for implementing functional precision medicine approaches. This opportunity is particularly relevant given growing evidence that BrM harbor additional layers of biology not captured by their primary tumors, with direct implications for therapeutic response and resistance (Xing et al, 2025).

A major unmet need in metastasis research is the coordinated acquisition of matched patient samples, ideally encompassing primary tumors, extracranial metastases, and brain lesions. In the context of BrM, this goal is especially challenging. Brain metastases can arise synchronously or metachronously, with latency ranging from months to decades, as frequently observed in breast cancer (Ho et al, 2015). Even when primary tumors are biopsied or resected, tissue availability is often limited or inaccessible due to limited coordination across clinical departments and institutions (Steindl et al, 2022). These constraints highlight the need for harmonized intra- and inter-institutional strategies to systematically collect and annotate patient-derived material for translational research.

### Availability of metastasis-focused cohorts, registries, and biobanks

Most existing BrM cohorts originate from single institutions, resulting in fragmented resources that require substantial effort to integrate into experimental pipelines. This fragmentation represents a major bottleneck for large-scale validation of laboratory findings and for addressing clinical heterogeneity. To partially overcome these limitations, RISEbrain has coordinated organ-specific cohorts across multiple clinical centers, including University Hospital Turin, Goethe University Hospital Frankfurt, and the Spanish RENACER network (Valiente and Ortega-Paino, 2024). This coordinated effort has enabled harmonization of clinical annotation, optimization of scarce resources such as tissue microarrays, and cross-cohort comparisons that would not be feasible within isolated datasets.

Despite these advances, access to well-annotated BrM cohorts remains uneven across Europe and beyond. Retrospective registries such as the Vienna Brain Metastasis Registry provide valuable long-term clinical data and archival samples, but reliance on fixed material limits functional and exploratory studies (Steindl et al, 2022). Prospective initiatives within the RISEBrain consortium and the Spanish RENACER network have expanded access to fresh surgical specimens, peripheral blood, and cerebrospinal fluid (CSF), enabling the generation of patient-derived models and multi-omic datasets. However, even these efforts face constraints related to sample availability, longitudinal follow-up, and regulatory barriers to data and model sharing.

### Practical challenges in generating data from clinical cohorts

Several operational challenges continue to limit the full potential of BrM clinical cohorts. First, standardized strategies for clinical data annotation and analysis are still lacking, complicating cross-study comparisons (Lin et al, 2015). Second, variability in tissue quality based on preoperative clinical parameters (Rombach et al, 2025). Third, tissue processing workflows—including fixation procedures, tissue microarray construction, single-cell dissociation protocols, and spatial assays—vary widely across centers, introducing technical variability (Rodriguez et al, 2024). Fourth, access to liquid biopsies, particularly CSF, is inconsistent and often restricted to specific clinical indications (Robinson et al, 2024). Together, these factors introduce logistical delays and potential biases that limit reproducibility and scalability.

**Figure 1:**
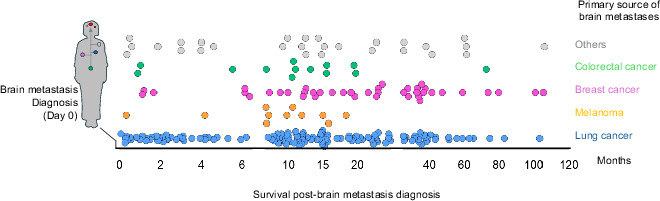
Survival heterogeneity among patients with brain metastases. Survival data from the most recent update (03.11.2025) of the RENACER cohort, a real-world multi-institutional effort (21 hospitals) initiated in 2021. The data depict overall survival (OS), defined as the time (in months) from the diagnosis of brain metastases to death from any cause, across brain metastasis (BrM) patients. To ensure adequate follow-up and minimize misclassification bias, we included only patients who had died or who were alive with at least 12 months of follow-up after BrM diagnosis. Patients who were alive with less than 12 months of follow-up were censored. In total, 236 patients fulfilled these criteria. Each dot is a patient.

These challenges are further exacerbated by the clinical course of BrM, which is characterized by limited survival in a substantial proportion of patients. Data from the RENACER cohort illustrate marked survival heterogeneity following BrM diagnosis, with many patients surviving less than six months, while others survive beyond 25 months, across primary cancer entities (Fig. 1). Detailed investigation of biological determinants underlying this heterogeneity requires large, well-annotated cohorts (Steindl et al, 2022). However, most published omics-based BrM datasets lack robust clinical annotation, precluding systematic correlations with outcomes such as overall survival (Xing et al, 2025) (Table 1). Although single-cell profiling of the BrM tumor microenvironment has expanded rapidly, most studies still fail to integrate clinical parameters, limiting insight into the functional contribution of specific cellular populations to patient prognosis (Table 1) (Biermann et al, 2022).

**Table 1:** Summary of available single-cell/single-nuclei RNA expression datasets from brain metastases.

Authors	Journal	Year	Cancer type	Data types	PMID
Bejarano et al, 2024	*Cancer Cell*	2024	Multiple	Single-cell transcriptomics	38242126
Bejarano et al, 2025	*Immunity*	2025	Multiple	Bulk, single-cell, and spatial transcriptomics	40107274
Biermann et al, 2022	*Cell*	2022	Melanoma	Single-cell and single-nucleus transcriptomics	35803246
Gonzalez et al, 2022	*Cell*	2022	Multiple	Single-cell transcriptomics	35063085
Jassowicz et al, 2026	*Cancer Cell*	2026	Breast	Bulk, single-nucleus, and spatial transcriptomics	42049023
Karimi et al, 2023	*Nature*	2023	Multiple	Proteomics	36725935
Kim et al, 2020	*Nature Communications*	2020	Lung	Single-cell transcriptomics	32385277
Klemm et al, 2020	*Cell*	2020	Multiple	Sorted bulk transcriptomics	32470396
Smalley et al, 2021	*Clinical Cancer Research*	2021	Melanoma	Single-cell transcriptomics	34035069
Song et al, 2023	*Communications Biology*	2023	Breast and lung	Single-cell transcriptomics	37479733
Tagore et al, 2025	*Nature Medicine*	2025	Lung	Single-nucleus transcriptomics	40016452
Wischnewski et al, 2023	*Nature Cancer*	2023	Multiple	Single-cell and bulk transcriptomics	37217652
Xing et al, 2025	*Cancer Cell*	2025	Multiple	Single-cell transcriptomics	40215980
Yang et al, 2026	*Nature Communications*	2026	Multiple	Single-cell and single-nucleus transcriptomics, proteomics, and WES	41639090
Zhang et al, 2022	*Nature Communications*	2022	Lung	Spatial transcriptomics	36216799

### Accessing early and non-surgically treated lesions

Non-surgically treated BrM constitute most clinically diagnosed lesions, yet they remain largely inaccessible for research. Depending on institutional practices, 60–100% of BrM do not undergo resection. These include lesions that are unsuitable for surgery due to location, size, or patient condition, irradiated metastases, surgical relapses, and lesions that remain stable or regress under systemic therapy. As a result, surgically derived cohorts are biased toward a subset of clinically accessible cases, potentially limiting translational relevance.

To address this gap, rapid autopsy programs represent a critical yet underutilized resource. Initiatives such as PEACE (Bailey et al, 2021), UPTIDER (Geukens et al, 2024), and BrainStorm (Martins-Branco et al, 2023) have demonstrated the feasibility and scientific value of coordinated autopsy-based research. These programs uniquely enable access to undiagnosed micrometastases, matched primary and metastatic sites, and, in some cases, pre-diagnosis blood or CSF samples. Importantly, they provide access to brain tissue without clinically detectable tumor burden, allowing investigation of early metastatic seeding and colonization—processes that remain difficult to model experimentally. Expansion of BrM-focused rapid autopsy programs, integrated within multinational consortia, will be essential for advancing secondary prevention strategies and for generating comprehensive datasets that capture the full metastatic cascade.

## Patient-derived models to boost translational efforts

Patient-derived materials are increasingly used not only as specimens for generating descriptive data (e.g., validating protein expression in human tissues), but also as functional platforms for generating actionable insights. For example, assessing the effect of an inhibitor in patient-derived organoids (PDOCs) can enable the identification of molecular signatures that stratify patients into likely responders and non-responders. A central goal in translational oncology is the development of patient “avatar” systems that can inform therapeutic decision-making. However, many established models, such as patient-derived xenografts, require extended expansion times, limiting their utility for real-time clinical applications.

### Patient-derived organotypic cultures as functional avatars

Patient-derived organotypic cultures (PDOCs) offer a promising way to overcome several of these limitations. PDOCs consist of thick sections of freshly resected tumor tissue that can be maintained ex vivo under optimized conditions for up to 7 days, preserving native tissue architecture and key microenvironmental interactions (Zhu et al, 2022). While PDOCs also have some constraints—such as a gradual decline in viability, activation of the glial compartment, and limited ability to recapitulate new immune recruitment—these aspects are well recognized and can be carefully considered when interpreting results (Valiente et al, 2025; Zhu et al, 2022). In BrM research, PDOCs have been widely applied for drug screening, prediction of therapeutic responses, and identification of predictive biomarkers (Steindl and Valiente, 2025). Moreover, their application in drug screening can be further strengthened by integrating complementary approaches—particularly when blood–brain barrier permeability is unknown, pharmacokinetic properties require optimization, or immune recruitment dynamics need to be more fully captured. A major advantage of this platform is speed: mechanistic insights can be generated within days, enabling timely assessment of patient-specific treatment sensitivity (Laue et al, 2026; Zhu et al, 2022).

**Figure 2:**
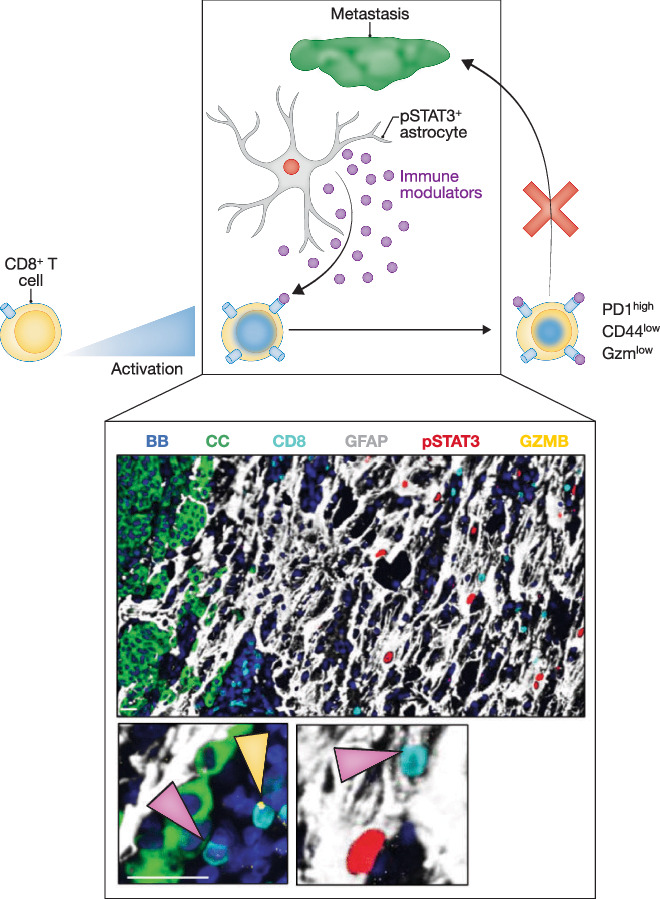
Schematic representation of the immunosuppressive axis between STAT3⁺ reactive astrocytes and CD8⁺ T cells in patients with brain metastasis. Figure adapted from Priego et al (Priego et al, 2025). Tumor-infiltrating CD8⁺ T cells undergo an initial activation that is subsequently restrained through interactions with immunosuppressive molecules (i.e., TIMP1) secreted by STAT3⁺ reactive astrocytes. This interaction results in a less activated, more exhausted CD8⁺ T cell phenotype characterized by reduced secretion of cytotoxic effector molecules, thereby facilitating tumor progression. Accordingly, therapeutic strategies designed to boost CD8⁺ T cell responses in brain metastasis are likely to require targeting astrocyte-dependent immunosuppression. The schematic is accompanied by a representative multiplex image from a brain metastasis patient sample, showing that STAT3⁺ reactive astrocytes are preferentially located in proximity to CD8⁺ T cells lacking granzyme B (GzmB) expression, whereas pSTAT3⁻ reactive astrocytes are found closer to GzmB⁺ CD8⁺ T cells. Higher magnification highlights both CD8⁺ granzyme B⁺ T cells (yellow arrows) and CD8⁺ granzyme B⁻ T cells (pink arrows). Scale bar, 20  μm.

In clinically relevant settings, PDOCs can be generated from surgical resections or biopsies and exposed to the same therapeutic regimens administered to patients within clinical trials. Histological and molecular evaluation of cancer cell proliferation, cell death, and microenvironmental responses provides early readouts of treatment efficacy that precede radiographic or clinical responses. This approach opens the possibility of stratifying patients into responder and non-responder groups early during treatment, thereby informing trial enrichment strategies and avoiding ineffective therapies (Álvaro-Espinosa et al, 2025; Laue et al, 2026; Priego et al, 2025; Zhu et al, 2022) (Fig. 2). Ongoing clinical trials are currently evaluating this paradigm (NCT05689619), although broader implementation will require additional regulatory and logistical frameworks to integrate functional testing into clinical workflows (Steindl and Valiente, 2025).

### Logistical and technical considerations

Despite their promise, PDOCs reliance on limited surgical material, finite slice viability, and restricted scalability naturally constrains their use for long-term studies, biobanking, and high-throughput drug screening. Nevertheless, experience within RISEbrain indicates that PDOCs remain a powerful and biologically faithful platform, even after prolonged tissue preservation, and can be shipped internationally as fresh material. Together, these features support centralized processing and the implementation of standardized operating procedures in specialized laboratories, thereby enhancing reproducibility and promoting equitable access across centers.

### Emerging modeling strategies

Emerging organoid-based and hybrid modeling strategies complement PDOCs by addressing these practical limitations while extending the experimental scope. Renewable induced pluripotent stem cells (iPSC)-derived brain organoids (Lancaster and Knoblich, 2014; Lancaster et al, 2013; Pasca et al, 2015) provide scalable and standardized platforms that can be co-cultured with disseminated cancer cells. As such, these self-organizing three-dimensional in vitro systems offer access to early metastatic seeding and dormancy stages that are not captured by resection-based PDOC models. Moreover, they enable large-scale compound screening and systematic perturbation studies that are difficult to achieve with scarce patient tissue. Human cortical organoid (hCO) co-cultured with cancer cells (Krieg et al, 2025) recently enabled the screening of over 300 compounds to identify selinexor as a potent inhibitor of melanoma brain metastatic viability, a scope of analysis often unfeasible with limited surgical explants. More advanced “avatar” approaches, such as embedding fresh patient tumor explants into cerebral organoids, retain key features of tumor heterogeneity and immune composition while supporting long-term culture and cryopreservation. For instance, the Individualized Patient Tumor Organoid (IPTO) model involves inserting fresh tumor explants into iPSC-derived cerebral organoids, which serve as a vascularized neural niche. This approach not only preserves the tumor’s immune microenvironment and spatial heterogeneity but also overcomes the temporal limitations of PDOCs by enabling long-term culture (up to 100 weeks) and biobanking via cryopreservation without loss of genomic fidelity (Peng et al, 2025).

**Figure 3:**
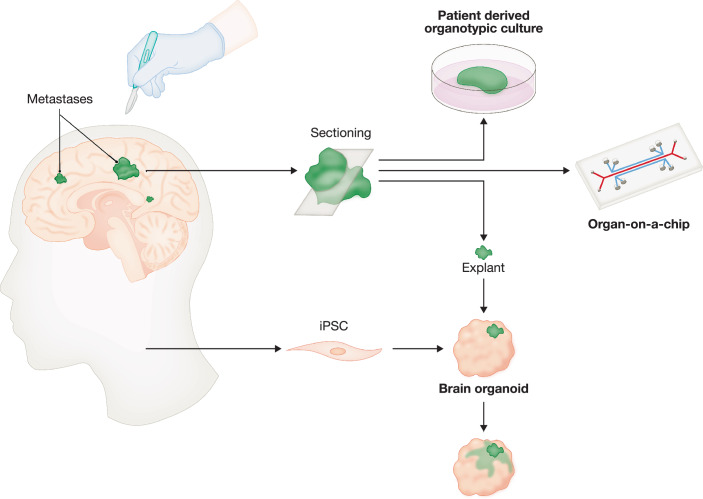
Patient-derived and engineered models for translational brain metastasis research. Patient tumor material obtained at surgical resection can be used to generate complementary ex vivo and in vitro model systems. Sectioned tumor tissue yields patient-derived organotypic cultures (PDOCs), or explants that can be embedded into iPSC-derived cerebral organoids for longer-term culture. In parallel, iPSCs can be differentiated into brain organoids and co-cultured with cancer cells to model early metastatic seeding and enable scalable drug screening. Both PDOC and organoid platforms can be further integrated into organ-on-a-chip devices to incorporate microfluidic flow and immune cell recruitment. Together, these approaches form a complementary toolkit bridging patient biology and experimental tractability.

While current brain organoids predominantly reflect embryonic or fetal brain states, ongoing advances—including vascularization (Kistemaker et al, 2025), microglia incorporation (Park et al, 2023), and computational modeling—are progressively increasing in their physiological relevance. Integration of microfluidic organ-on-chip (Papamichail et al, 2025) and patient-derived immune cells that can circulate and infiltrate the tumor tissue will further enable new research directions exploring cross-organ communication, an emerging topic in BrM (Massara et al, 2025). Overall, PDOCs and organoid-based platforms form a complementary toolkit: PDOCs preserve key features of the patient tumor microenvironment, including stromal and immune components present in the explanted tissue, whereas organoids offer advantages in scalability, temporal resolution, and access to otherwise inaccessible stages of BrM (Fig. 3).

## Data processing and harmonization from bulk to single-cell transcriptomics

Transcriptomic profiling has become a cornerstone of BrM research, enabling systematic characterization of tumor and microenvironmental states. Both bulk and single-cell RNA sequencing approaches offer distinct and complementary advantages, and their integration is increasingly recognized as essential for translational impact.

### Value of bulk RNA sequencing in BrM research

Bulk RNA sequencing remains a resource-efficient and robust approach for generating reproducible transcriptomic data across large patient cohorts. Its scalability, cost-effectiveness, and relative technical stability make it particularly well-suited for multi-center studies, where tissue availability and processing conditions vary (Lu et al, 2025). In the context of BrM, bulk RNA-seq mitigates dissociation-induced biases that disproportionately affect fragile neural and stromal populations and enables inclusion of larger sample sizes than currently feasible with single-cell technologies.

Bulk RNA-seq has proven especially valuable for biomarker discovery, tumor classification, and correlation of molecular states with clinical parameters. Deconvolution algorithms such as CIBERSORT (Newman et al, 2015) have extended the utility of bulk RNA-seq data by enabling computational inference of cell-type composition from mixed-tissue profiles, partially bridging the resolution gap with single-cell approaches. Its lower susceptibility to technical dropout and batch effects facilitates harmonization across institutions and sequencing platforms, enabling integrative analyses of heterogeneous cohorts (Dai et al, 2026). These features are critical for capturing the clinical diversity of BrM arising from different primary tumor types and brain niches (Yang et al, 2026). Collectively, bulk RNA-seq provides the statistical power and cohort depth necessary to identify clinically actionable transcriptomic signatures, complementing the higher cellular resolution offered by single-cell and spatial approaches.

### Single-cell and single-nucleus transcriptomics

Single-cell RNA sequencing has transformed cancer research by resolving complex tissues into their constituent cellular populations and transcriptional states. In BrM, scRNA-seq studies have provided key insights into the composition of the microenvironment, the potential tumor–microenvironment interactions, immune infiltration patterns, and intratumoral heterogeneity (Gonzalez et al, 2022; Kim et al, 2020). However, logistical and technical constraints limit widespread application. Fresh tissue requirements restrict sample availability, and enzymatic dissociation underrepresents neurons, astrocytes, and vascular cells.

To address these limitations, single-nucleus RNA sequencing has gained traction, enabling the profiling of frozen as well as formalin-fixed and paraffin-embedded BrM samples and an improved capture of brain-resident cell populations (Wang et al, 2024). Nevertheless, these advantages of snRNA-seq come at a cost: as nuclei lack cytoplasmic RNA, the resulting libraries contain fewer transcripts and are preferentially depleted of transcripts localized outside the nucleus. This reduced coverage translates into sparser count matrices and a weaker and noisier signal in many analyses, including the detection of subtle cell states and the inference of chromosomal copy-number variations, compared with single-cell approaches. Thus, snRNA-seq analysis is often challenging, does not always recapitulate observations from scRNA-seq, and the results must be interpreted with caution.

### Approaches to integrating disparate datasets in the context of brain metastases

Harmonization and integration strategies have become central approaches for overcoming the limited size and fragmentation of individual cohorts in BrM research. By combining datasets generated across studies, institutions, and platforms, researchers aim to increase statistical power and capture broader clinical and biological diversity. However, such integration is inherently challenging, particularly when datasets are generated using different library preparation protocols, sequencing depths, and technological platforms. Methods such as scVI (Lopez et al, 2018) and Harmony (Korsunsky et al, 2019), are widely used to mitigate batch effects and align shared transcriptional structure across datasets.

It is important to note, however, that these methods may not be able to remove all batch effects and that they carry a risk of overcorrection. In heterogeneous diseases such as BrM—where differences in primary tumor origin, metastatic niche, treatment history, and microenvironmental composition drive genuine biological diversity—batch correction can inadvertently remove or dampen true biological signals. As a result, integration can lead to artificial homogenization of distinct tumor states, masking clinically relevant differences and potentially biasing downstream analyses such as subtype classification, biomarker discovery, or trajectory inference. The challenge, therefore, lies in distinguishing technical noise from biologically meaningful variation, particularly when these sources of variation are partially confounded. As an alternative strategy, some studies focus initially on within-sample variability, thereby reducing reliance on cross-cohort harmonization, and then aggregate the results across all analyzed samples (Gavish et al, 2023).

### Integrative strategies and computational deconvolution

Given these complementary strengths and limitations, integrative strategies that bridge bulk and single-cell data are essential. Single-cell and single-nucleus atlases provide high-resolution reference maps of tumor, immune, and stromal cell states that can be leveraged to computationally deconvolute bulk transcriptomes (Gonzalez et al, 2022; Kim et al, 2020). Deconvolution approaches enable inference of cell-type proportions and state-specific expression programs within large, clinically annotated bulk datasets, effectively linking mechanistic insights to clinical scale (Nguyen et al, 2024). Selection of reference single-cell datasets for bulk deconvolution remains a key challenge, as scRNA-seq data are more directly comparable to bulk profiles but often lack key brain-resident populations, whereas snRNA-seq captures broader cellular diversity at the cost of technical comparability. Addressing this balance will be critical for robust application of deconvolution methods and for translating transcriptomic insights into clinically meaningful predictions.

## Brain metastasis spatial transcriptomics and proteomics: the path forward

The tumor microenvironment (TME) of BrM constitutes a hybrid ecosystem that integrates features of the primary tumor with unique properties of the central nervous system, including the blood–brain barrier (Quail and Joyce, 2017). This spatial complexity cannot be fully captured by bulk or dissociative single-cell analyses alone (Biermann et al, 2022; Gonzalez et al, 2022; Kim et al, 2020; Xing et al, 2025). High-dimensional spatial transcriptomics and proteomics imaging platforms now enable multiplexed RNA and protein mapping while preserving tissue architecture, offering unprecedented insight into the organization and function of the metastatic niche (Liu et al, 2026).

### Current spatial transcriptomics approaches

Spatial transcriptomics maps gene expression within the spatial context of intact tissue, preserving native cellular architecture and local neighborhoods—a feature particularly valuable for examining tumor heterogeneity, cellular interactions, and how these shape disease progression and therapeutic response (See et al, 2025). These technologies are broadly categorized into imaging-based methods, where target identity is linked to fluorescent signals through multiplexed hybridization (e.g., Xenium [10x Genomics], CosMx SMI [NanoString], MERSCOPE [Vizgen]) and sequencing-based approaches, where spatial coordinates are linked to transcripts via spatial barcoding and sequencing (e.g., Visium HD [10x Genomics], Stereo-seq [STOmics]). Imaging-based platforms are capable of detecting hundreds to thousands of RNA targets directly in tissue with subcellular resolution, often with the ability to profile proteins as well. Sequencing-based technologies like Visium HD use spatially barcoded microarrays to provide unbiased, whole-transcriptome coverage at high spatial fidelity, achieving single-cell resolution across a defined capture area. Emerging sequencing technologies such as Stereo-seq extend this further with ultra-high-resolution maps across larger tissue areas. In parallel, newer systems like G4X Spatial Sequencer (Singular Genomics) aim to integrate spatial in situ sequencing of RNA with proteomics and morphology on the same FFPE section at subcellular resolution and high throughput—offering multimodal readouts from the same sample. Notably, most spatial transcriptomics technologies can now be broadly deployed on both formalin-fixed paraffin-embedded (FFPE) and fresh-frozen tissues (e.g., Visium HD, Xenium, CosMx SMI, MERSCOPE) with great success, as reliable protocols exist for both human and mouse tissues (Jung and Kim, 2023). These strategies have been thoroughly reviewed recently (Liu et al, 2026). In the context of brain metastasis research, spatial transcriptomics has begun to shed light on the complexity and diversity of the metastatic microenvironment, revealing distinct niches and signaling circuits that drive metastatic progression and response to therapy (Li et al, 2025; Manukyan et al, 2025; Zhang et al, 2022). To gain a more granular and refined definition of cell states and neighborhoods, unbiased (whole transcriptome) cost-intensive spatial omics profiling technologies such as Visium HD are frequently combined with targeted omics strategies, such as custom Xenium-designed panels, for in-depth analysis of selected regions across biological samples (Liu et al, 2026). Moreover, the field is rapidly moving towards integrating spatial transcriptomics with other omics approaches (e.g., proteomics, [epi]-genomics, metabolomics) and advanced hiplex imaging modalities to gain a holistic understanding of the brain metastatic ecosystem (Yang et al, 2026). Taking such an approach, Yang et al, revealed a novel mechanism where epithelial-mesenchymal transition (EMT) programs facilitate immune infiltration within the brain microenvironment, challenging conventional views and providing a framework for precision medicine.

### Current spatial proteomics approaches

Technologies such as imaging mass cytometry (IMC) and multiplex immunohistochemistry (mIHC) have been successfully applied to BrM, revealing spatial patterns of immune infiltration, tumor–stromal interactions, and endothelial associations that support metastatic colonization (Karimi et al, 2023). IMC enables simultaneous detection of dozens of protein markers at subcellular resolution through metal-tagged antibodies and laser ablation, allowing reconstruction of high-dimensional spatial maps. Studies applying IMC to BrM have demonstrated marked differences in immune infiltration across tumor types and highlighted perivascular tumor–endothelial interactions as a conserved feature of metastatic outgrowth (Karimi et al, 2023).

Multiplex immunofluorescence (MxIF) approaches based on iterative staining and signal amplification, such as OPAL™-Tyramide Signal Amplification (TSA), provide complementary spatial information on FFPE material and are compatible with diagnostic pathology workflows (Biermann et al, 2022). Applications of these technologies to melanoma and lung cancer BrM have revealed spatial segregation of immune populations, neuronal-like tumor phenotypes, and distinct cytokine networks within the metastatic niche (Biermann et al, 2022). More recently, their application to primary brain tumors has identified tertiary lymphoid structures (TLSs) with active immune features, which are associated with improved overall survival (Cakmak et al, 2025). Such structures are also of high interest in brain metastasis, and their response to immunotherapy (Jassowicz et al, 2026).

Although ST- and MxIF-based approaches are increasingly applied to BrMs, this is not reflected in the adoption of non-antibody-based spatial proteomics approaches, such as laser capture microdissection coupled to LC–MS/MS or MALDI-based mass spectrometry imaging. To date, no study has applied such methodologies to human BrM samples. This led to a proteomic characterization of BrMs landscape exclusively through bulk mass spectrometry workflows. For instance, Yang et al (Yang et al, 2026) constructed the largest pan-cancer multi-omic atlas of 1032 BrMs, identifying four molecular subtypes with distinct biological programs and therapeutic vulnerabilities, and validated these transcriptomic subtypes at the protein level through quantitative 4D-DIA proteomics on 118 BrMs and targeted metabolomics on 74 BrMs. Similarly, Yang et al (Yang et al, 2025) performed multidimensional proteomics and phosphoproteomics on 60 BrMs from different entities. Comparing their proteomic profile with that of glioma samples highlighted distinct PI3K-Akt pathway activation patterns and potential plasma biomarkers.

### Technical and analytical challenges

Despite rapid progress, spatial proteomics faces several technical and analytical challenges. Panel design is complicated by antibody cross-reactivity, shared marker expression across multiple cell types, and signal overlap, necessitating careful optimization and validation. In parallel, high-plex imaging generates large, complex datasets that require advanced computational infrastructure for storage, processing, and analysis. Accurate cell segmentation remains particularly challenging in brain tissue due to irregular morphology and background signal; however, emerging deep-learning approaches are increasingly able to improve segmentation accuracy while reducing manual annotation requirements (Lee et al, 2025; Pang et al, 2025). Compared with IMC, iterative fluorescence-based approaches such as cyclic immunofluorescence and OPAL^TM^-TSA mIHC offer higher optical resolution, faster scanning, larger imaging areas, and greater flexibility in antibody selection, but are more affected by autofluorescence, spectral overlap, and repeated staining-related tissue degradation. In contrast, IMC enables single-shot detection of more than 40 markers using metal-tagged antibodies and laser-ablation mass spectrometry with minimal background; however, at a slower speed, in a more ROI-focused manner, and with destructive image acquisition (Semba and Ishimoto, 2024). Lastly, while current studies using non-antibody based protemoics appraoches offer increased depth of proteome coverage, these inherently lack the spatial resolution needed to resolve intratumoral heterogeneity and region-specific molecular programs within the BrM microenvironment.

### Toward integrative spatial atlases

A holistic understanding of BrM will require coordinated efforts to generate large, multi-center datasets that integrate multi-level spatial omics data with genetic, histological, and clinical information. Given the pronounced spatial heterogeneity of BrM and the routine availability of H&E sections, applying histology-based machine learning to spatial omics represents a promising, scalable, and clinically relevant avenue. Ultimately, these approaches are poised to move beyond descriptive mapping toward predictive frameworks that inform therapy selection, identify resistance mechanisms, and reveal actionable vulnerabilities within the metastatic brain niche (Yang et al, 2026).

## Towards clinical translation of liquid biopsies for brain metastases

One of the major challenges in metastasis research is the inability of conventional imaging modalities, such as MRI, to detect the earliest stages of metastatic dissemination and colonization. Liquid biopsies (LBs) offer a minimally invasive approach to capture molecular information from otherwise inaccessible disease stages and to longitudinally monitor tumor evolution and treatment response in real time.

### Opportunities and challenges in liquid biopsy approaches

Liquid biopsies based on the analysis of circulating tumor DNA (ctDNA), circulating tumor cells (CTCs), extracellular vesicles (EVs), and proteins from biofluids such as blood, CSF, and urine provide complementary information to tissue biopsies (Dwarshuis et al, 2025). A major challenge with liquid biopsies and biomarker discovery in BrM is the biological and anatomical isolation of intracranial tumors from the systemic circulation. The blood–brain barrier (BBB) and blood–tumor barrier (BTB) significantly limit the release of tumor-derived material—such ctDNA, RNA, proteins, and EVs—into peripheral blood. As a result, blood liquid biopsies often show low sensitivity in BrM patients. CSF-based liquid biopsies offer improved sensitivity and brain specificity, as it more directly reflects biological processes occurring within the central nervous system and may capture tumor-derived biomarkers that are undetectable or absent in peripheral blood (De Mattos-Arruda et al, 2015) (Table 2). Notably, minimally invasive temporal monitoring through liquid biopsies enables personalized tracking of disease progression and therapeutic response and offers a potential window into the clinically invisible phase of metastatic outgrowth (Nakasu et al, 2023; Rubio-Perez et al, 2021). Nevertheless, integration of CSF-based LB approaches into routine clinical practice remains constrained by technical, logistical, and regulatory challenges. In particular, the invasive nature of CSF collection, limited standardization, and reduced feasibility for frequent serial sampling hinder widespread adoption (Bagley et al, 2025).

**Table 2:** Emerging brain metastases biomarkers.

Authors	Journal	Year	Cancer type	Data types	PMID
Priego et al, 2025	*Cancer Discovery*	2025	Multiple	Bulk and single-cell transcriptomics, proteomics	39354883
Alvaro-Espinosa et al, 2026	*Cancer Research*	2026	Multiple	Bulk and single-cell transcriptomics, proteomics	41874311
Monteiro et al, 2022	*Nature Medicine*	2022	Multiple	Bulk and single-cell transcriptomics, proteomics	35411077
Adler et al, 2023	*Nature Cancer*	2023	Lung and melanoma	Proteomics	36797502
Zuccato et al, 2025	*Nature Medicine*	2025	Lung	Cell-free DNA methylomics, tissue methylomics	39379704
De Mattos-Arruda et al, 2015	*Nature Communications*	2015	Lung and breast	Targeted sequencing, WES, and ddPCR	26554728

In addition, the reliable detection of low-abundance biomarkers within complex biofluids requires ultra-sensitive and highly specific analytical platforms, and no universal standards currently exist for sample processing, normalization, or analysis (Colapietro et al, 2025). All these considerations have been key to understand the lack of clinical translation of existing biomarkers reported in the bibliography (Table 2) (Yu et al, 2025).

### Emerging photonic and point-of-care technologies

In addition to established approaches to score biomarkers in liquid biopsies (e.g., ELISA, cfDNA), recent advances in photonic biosensors have introduced fluorescence-free platforms capable of real-time, high-sensitivity detection of circulating biomarkers using minimal sample volumes (Barth and Lee, 2025). The recent development of metamaterial-based photonic sensors offers rapid readouts, ultra-low detection limits, and is compatible with complex liquid biopsy samples, aligning with the demands of personalized oncology (Koc et al, 2024). These tiny engineered optical structures detect extremely small physical, chemical, or biological changes by measuring how they alter the way light resonates within the material. Rather than converging on a single diagnostic modality, the field is increasingly moving toward functional complementarity, integrating diverse optical and molecular platforms tailored to specific clinical contexts (Altug et al, 2022).

Successful clinical translation of these technologies will depend on sustained interdisciplinary collaboration between oncology, bioengineering, and photonics, as well as on the development of portable, cost-effective point-of-care devices that enable continuous monitoring and real-time therapeutic adaptation (Carrara et al, 2026).

## Closing remarks and future perspectives

Patient-derived resources are reshaping BrM research by enabling direct interrogation of human disease at molecular, spatial, and functional levels (Gonzalez et al, 2022; Kim et al, 2020). Integration of clinical cohorts, ex vivo and in vitro models, multi-scale omics, spatial proteomics, and liquid biopsy technologies provides a foundation for earlier detection, secondary prevention, and more precise therapeutic strategies (Valiente et al, 2025).

Realizing this potential will require sustained coordination across clinical and research centers, standardized workflows for tissue and data processing, secure frameworks for data and model sharing, and close alignment between exploratory research and clinical practice. Importantly, such efforts are part of the roadmap for the European Health space (Nikolski et al, 2024). As these infrastructures mature, they are expected to transform our understanding of brain metastasis ecosystems and accelerate the translation of biological insight into tangible clinical benefit, particularly for patients at high risk of brain involvement or therapy resistance.

## Supplementary information

Peer Review File
