# Supplementary material for: Patient-derived resources for decoding and targeting brain metastases ecosystems
Source: EMBO Mol Med. 2026 Jul 3;18(8):3017–27. doi: 10.1038/s44321-026-00472-y (PMC13470209; doi:10.1038/s44321-026-00472-y)
Supplement: Supplementary file 1 [file 44321_2026_472_MOESM1_ESM.pdf]

EMBO Molecular Medicine

Patient-Derived Resources for Decoding and Targeting Brain Metastases Ecosystems

Manuel Valiente, Aydoğan Era Lal, Nisan Feigin, Clara Gahn, Caroline Hernandez-Oliver, Friedrich Milling, evren oktem, Carmen Ortega-Sabater, Neibla Priego, Alessia Ricci, Giulia Capella, Luca Mangherini, Laura Serrano, Antonia Stammberger, Juan Vazquez-Canto, Katharina Weber, Rebekka Wehner, Thomas Broggini, Marcus Czabanka, Serap Aksu, Fátima Al-Shahrour, Luca Bertero, Marc Schmitz, Itay Tirosh, and Hind Medyouf

Corresponding authors: Hind Medyouf (hmedyouf@ukaachen.de) , Manuel Valiente (mvaliente@cnio.es)

Review Timeline:

Submission Date:	22nd Feb 26
Editorial Decision:	10th Apr 26
Editor's Follow-up Email:	13th Apr 26
Revision Received:	20th May 26
Editorial Decision:	21st May 26
Revision Received:	22nd May 26
Accepted:	22nd May 26

Editor: Lise Roth

Transaction Report:

(Note: With the exception of the correction of typographical or spelling errors that could be a source of ambiguity, letters and reports are not edited. Depending on transfer agreements, referee reports obtained elsewhere may or may not be included in this compilation. Referee reports are anonymous unless the Referee chooses to sign their reports.)

10th Apr 2026

Dear Prof. Medyouf, Dear Hind,

Thank you for submitting your perspective to EMBO Molecular Medicine. Please accept my apologies for the delayed response as I was waiting for a referee report. Unfortunately, referee #3 has not yet provided feedback. However, as the other two referees are very positive, I would like to make a decision based on the reports I have.

As you will see below, both referees acknowledge your article's value to the community. They also offer suggestions to enhance the interest and impact of your work. We would therefore welcome a revised version of your manuscript that addresses these points. If referee #3 provides a report shortly, we will forward it to you and ask that you address this referee's comments as well, understanding that they will be minor.

Please attach a covering letter detailing how you have addressed each point raised by the referees.

Additionally, please address the following editorial matters:
1. Please upload the manuscript text in .docx or .rtf format and remove the figures from the manuscript text.
2. Authors: please spell out the authors' first names, followed by last names; they should appear exactly as they should be published.
3. Acknowledgements/Funding: the information provided in the manuscript should match the information provided in the submission system. Should TRANSCAN-3/European Commission and the Else Kroener Fresenius Foundation be included in the list of funders in our system?
4. Please provide a Disclosure and Competing Interests statement.
5. Please include a list of the consortium members, including names and affiliations.

For the figures, please note:
1. The figures should be uploaded as separate, high resolution figure files. Please compile the legends at the end of the manuscript text.
2. Perspectives do not have Materials and Methods, and thus cannot contain new scientific figures. Please use already published figures or illustrative graphs.
3. If the figures or part of the figures have been adapted from a published figure, please add this information to the figure legend (e.g., 'Adapted from...' or 'Based on...').
4. Each figure should be referenced in the text (currently, a callout is missing for Figure 2).

Looking forward to receiving your revised manuscript at your earliest convenience.

With kind regards,

Lise

Lise Roth, Ph.D
Senior Editor
EMBO Molecular Medicine

***** Reviewer's comments *****

Referee #1 (Remarks for Author):

The article convincingly describes the complexity of studying brain metastases, particularly with regard to the availability of appropriate patient material and the associated clinical data. In addition, it provides a helpful overview of several current initiatives, including the RISEBrain Consortium, the Spanish RENACER network, PEACE, UPTIDER, and BrainStorm. This

overview creates important transparency regarding existing research efforts. Such an overview is highly valuable, as it may facilitate future collaboration and the shared use of data and biological materials across research groups.

In particular, the survival data from the most recent update (03 November 2025) of the RENACER cohort (N = 236) represent a noteworthy resource. This real-world, multi-institutional effort involving 21 hospitals and initiated in 2021 provides valuable clinical data that could significantly support future research in this field.
In this context, I would like to suggest a minor addition to the manuscript. A table summarizing available RNA expression datasets (e.g., bulk RNA-seq, spatial transcriptomics, etc.) would be a valuable complement to the article. Such a resource would further increase the utility of the manuscript for readers by facilitating access to relevant datasets and reducing the need for time-consuming independent searches for suitable data resources.

Referee #2 (Remarks for Author):

Valiente et al. proposes a well-organized and relevant resume of the current perspective in brain metastasis cancer research, focusing of patient-derived resources. They review the novel models, technics and their limitations. This is an interesting perspective article that will be valuable to the community.
Here are some suggestions and comments to improve the manuscript.
1. Figure 1. The definition of the abbreviation CRC is missing
2. A more detailed description of the novel models would be valuable. I would suggest adding a diagram depicting the models described.
3. The bulk and single-cell transcriptomics section fells short and less detailed compared to the spatial transcriptomic section. Expanding a bit on this section would improve the manuscript.

Dear Hind, Dear Manuel,

Following my decision on April 10, I received the report from referee #3 over the weekend (attached). As you will see, like referees #1 and #2, they recognize the value of the perspective but offer several suggestions for improvement. We find these suggestions valuable and would like you to address them, along with the comments from referees #1 and #2, in your revised manuscript and point-by-point rebuttal letter.

Please accept my renewed apologies for the delay in sending you the last report.

If you would like to discuss the points raised by the referees further, I am available to do so via email or video.

With kind regards,

Lise

Referee #3 – Remarks for authors

In this Perspective, the authors briefly describe current advancements and remaining challenges in the implementation of patient material for studying brain metastasis. The paper is well structured and oBers relevant and insightful discussion points on the model development, application of transcriptomic approaches and spatial technologies, use of liquid biopsies. Nonetheless, the text lacks depth for covering these topics, and has multiple overstatements. Here, we provide further feedback, which we hope will help to substantially improve the current version.

Major comments:

- Multiple parts of the review appear quite superficial. While mentioning issues, the authors do not specify and elaborate these. For people working on similar projects and closely with clinicians it can sound very familiar and probably clear, but for people outside of this niche, it will not. For instance, one can elaborate on "logistical delays and potential biases" in paragraph 3 page 3.

- Figure 1 requires better representation of the data. For instance, in a histogram format and without breaking the axis. It also can be useful to discuss in which cancer types there are more long-term versus short-term survivors. Why is it the case?

- In the page 5 top paragraph there is a statement about brain slices "preserving tissue architecture and microenvironmental interactions" which is an oversimplification misrepresenting a more nuanced reality. Organotypic slices are very useful tools but not without drawbacks, such as a substantial apoptotic and necrotic cell death (Staal 2011 https://journals.plos.org/plosone/article/file?id=10.1371/journal.pone.0022040&type=printable), glial scar formation (Grabiec 2017

https://pmc.ncbi.nlm.nih.gov/articles/PMC5614375/pdf/jove-126-55359.pdf), and, in case of using serum free culturing of organotypic slices, epileptiform activity (Magalhães 2018 https://link.springer.com/content/pdf/10.1186/s12974-018-1225-2). In addition, the authors do not discuss how long these samples can be used ex vivo.

- In the page 5 second paragraph, it is not mentioned what are the limitations of testing clinical trials drugs on PDOCs. For instance, absence of conditions mimicking BBB permeability and diBiculty to mimic pharmacodynamics similar to in vivo.

- The section "Emerging modelling strategies" requires mentioning of the lack of adaptive immune system in the PDOCs. And such statement as "PDOCs provide unmatched fidelity to the patient tumor microenvironment" is an overstatement, please adjust it.

- In "Single-cell and single-nucleus transcriptomics" section it is not mentioned what can be the caveats of snRNAseq, for instance, access to the good reference data.

- It is unclear why the authors focus on spatial transcriptomics as a tool to characterize tumor microenvironment. While it is a current trend to implement these

commercialized technologies, it is important to highlight publications which benchmark those and mention other, more aBordable techniques, which can be informative for patient samples at levels other than transcriptome. For instance, non-antibody based spatial proteomics (LCM LC-MS, MALDI) and spatial metabolomics, lipidomics (MALDITOF).

- The authors mention IMC and iterative staining approaches. It will be more informative to briefly compare these methods. There are critical diBerences, such as availability of metal-conjugated antibodies, size of scanning area, tissue damage, resolution, scanning time.

- Lastly, sections discussing liquid biopsies are also very descriptive, lacking details on important points. Are there any biomarkers for following brain metastases? If they can't be measured with routine methods, can we learn from other fields (eg. neurodegeneration)?

Apart from the discussed biosensors, authors could mention applicability of other methods (colorimetric, fluorescence-based assays, ELISA) and developments of sensitive proteomics workflows already implemented for clinical trial samples, such as SISCAPA method (Mabrouk OS.2020

https://www.frontiersin.org/journals/neuroscience/articles/10.3389/fnins.2020.00526/full).

Minor comments:

- The review process will benefit from the line numbering to navigate the text.
- Sections titles lack clarity. For example, "Exploiting liquid biopsies for brain metastasis" does not specify the purpose.
- In the statement in page 3 last paragraph, which combines information about rapid autopsy programmes. Are the authors sure that their statement regarding sample access is accurate for all the programmes mentioned? As, for instance, UPTIDER does not directly have access to matched primary tumors and metastases, but it is possible to retrieve archived tumor samples from pathology department (mentioned in Guekens T. et al, 2024).
- What are called "functional data" in page 5 top paragraph?
- Placement of some references needs to be adjusted. In particular, in "Emerging modelling strategies" section - in the first sentence references should be in the end of the sentence and (Krieg et al, 2025) reference should be placed prior comma. Same applies to (Wang et al., 2024) later in the text.

Point-by-point response to reviewer's comments

Referee #1 (Remarks for Author):
The article convincingly describes the complexity of studying brain metastases, particularly withregard to the availability of appropriate patient material and the associated clinical data. Inaddition, it provides a helpful overview of several current initiatives, including the RISEBrainConsortium, the Spanish RENACER network, PEACE, UPTIDER, and BrainStorm. This overviewcreates important transparency regarding existing research efforts. Such an overview is highlyvaluable, as it may facilitate future collaboration and the shared use of data and biologicalmaterials across research groups. In particular, the survival data from the most recent update (03 November 2025) of theRENACER cohort (N = 236) represent a noteworthy resource. This real-world, multi-institutionaleffort involving 21 hospitals and initiated in 2021 provides valuable clinical data that couldsignificantly support future research in this field.
In this context, I would like to suggest a minor addition to the manuscript. ***A table summarizingavailable RNA expression datasets (e.g., bulk RNA-seq, spatial transcriptomics, etc.) would be avaluable complement to the article.*** Such a resource would further increase the utility of themanuscript for readers by facilitating access to relevant datasets and reducing the need fortime-consuming independent searches for suitable data resources.

We would like to warmly thank reviewer#1 for the positive and thoughtful evaluation of our manuscript. We appreciate the recognition of the effort to highlight the complexity of studying brain metastases, as well as the value of summarizing current collaborative initiatives and datasets. The comments regarding the transparency and utility of these resources are very encouraging. We are also grateful for the constructive suggestion to include a summary of available single cell/single nuclei RNA expression datasets. In response, we have added a new **Table 1**, which compiles relevant transcriptomic resources, including single cell, bulk RNA-seq and spatial transcriptomics datasets. We hope this addition enhances the practical value of the manuscript by facilitating access to key data and reducing the need for independent dataset searches. In addition, we have further expanded the presentation of the RENACER cohort data. Specifically, we now provide survival data stratified by primary disease entity, which we believe improves both the interpretability and usability of this resource for the research community. We thank again reviewer#1 for the helpful feedback, which has strengthened the manuscript.

Referee #2 (Remarks for Author):
Valiente et al. proposes a well-organized and relevant resume of the current perspective in brainmetastasis cancer research, focusing of patient-derived resources. They review the novel models, technics and their limitations. This is an interesting perspective article that will be valuable to the community. Here are some suggestions and comments to improve the manuscript.
1. Figure 1. The definition of the abbreviation CRC is missing
This point has been addressed in the new Figure 1. Thank you for pointing it out.

2. A more detailed description of the novel models would be valuable.
I would suggest adding a diagram depicting the models described.
This section has been edited as per reviewer's request. Additionaly, a schemtic view of the emerging models discussed is now depicted in Figure 3, which is referenced in p5 line 206.

3. The bulk and single-cell transcriptomics section fells short and less detailed compared to the spatial transcriptomic section. Expanding a bit on this section would improve the manuscript.

We thank the reviewer for this constructive comment. We agree that the bulk and single-cell transcriptomics section warranted further elaboration to match the depth of the spatial transcriptomics discussion. We have expanded this section, and we now relate to two additional aspects. First, we mention computational deconvolution approaches, which partially bridge the resolution gap between bulk and single-cell methods by enabling inference of cell-type composition from mixed-tissue profiles. Second, we added a discussion about approaches to integrating disparate datasets in the context of brain metastases. We believe that these revisions improve the balance between the sections.

Referee #3 – Remarks for authors

In this Perspective, the authors briefly describe current advancements and remaining challenges in the implementation of patient material for studying brain metastasis. The paper is well structured and offers relevant and insightful discussion points on the model development, application of transcriptomic approaches and spatial technologies, use of liquid biopsies. Nonetheless, the text lacks depth for covering these topics, and has multiple overstatements. Here, we provide further feedback, which we hope will help to substantially improve the current version.

Major comments:

- Multiple parts of the review appear quite superficial. While mentioning issues, the authors do not specify and elaborate these. For people working on similar projects and closely with clinicians it can sound very familiar and probably clear, but for people outside of this niche, it will not. For instance, one can elaborate on "logistical delays and potential biases" in paragraph 3 page 3.

Thank you for this thoughtful and constructive comment. We agree that parts of the original text were too general and did not sufficiently elaborate on key limitations and practical considerations. In response, we have revised the section substantially to improve clarity and depth. Specifically, we removed overly broad statements and expanded the discussion of PDOCs to more clearly outline both their strengths and limitations. The revised text (page 4, line 192) now explicitly addresses constraints such as limited culture duration due to viablity, glial activation, and incomplete modeling of immune recruitment, and emphasizes how these factors should be considered when interpreting results. In addition, we provide more concrete context regarding their application in drug screening and highlight scenarios where complementary approaches are needed. Overall, these revisions aim to make the discussion more informative and accessible to readers outside the immediate field while maintaining relevance for specialists.

- Figure 1 requires better representation of the data. For instance, in a histogram format and without breaking the axis. It also can be useful to discuss in which cancer types there are more long-term versus short-term survivors. Why is it the case?

Thank you for this helpful suggestion. In response, we have replaced the original figure with a new version that improves the clarity and representation of the data. Specifically, we now include an updated figure based on the RENACER dataset, which more clearly illustrates brain metastasis (BrM) survival across primary disease entities. We believe this revised visualization addresses the concerns regarding data presentation. In addition, rather than speculating on differences between cancer types, we have included a statement noting that “detailed investigation of the biological determinants underlying this heterogeneity requires large, well-annotated cohorts,” to more appropriately frame this point.

- In the page 5 top paragraph there is a statement about brain slices "preserving tissue architecture and microenvironmental interactions" which is an oversimplification misrepresenting a more nuanced reality. Organotypic slices are very useful tools but not without drawbacks, such as a substantial apoptotic and necrotic cell death (Staal 2011. https://journals.plos.org/plosone/article/file?id=10.1371/journal.pone.0022040&type=printable), glial scar formation (Grabiec 2017 https://pmc.ncbi.nlm.nih.gov/articles/PMC5614375/pdf/jove-126-55359.pdf), and, in case of using serum free culturing of organotypic slices, epileptiform activity (Magalhães 2018 https://link.springer.com/content/pdf/10.1186/s12974-018-1225-2). In addition, the authors do not discuss how long these samples can be used ex vivo.

- In the page 5 second paragraph, it is not mentioned what are the limitations of testing clinical trials drugs on PDOCs. For instance, absence of conditions mimicking BBB permeability and difficulty to mimic pharmacodynamics similar to in vivo.

- The section "Emerging modelling strategies" requires mentioning of the lack of adaptive immune system in the PDOCs. And such statement as "PDOCs provide unmatched fidelity to the patient tumor microenvironment" is an overstatement, please adjust it.

Thank you for these constructive comments and suggestions. In response, we have revised the text to provide a more balanced and nuanced description of PDOCs. Specifically, we now clarify the limited *ex vivo* culture duration (up to ~7 days under optimized conditions) and explicitly acknowledge key constraints, including gradual declines in viability, alterations in the glial compartment, and the lack of de novo immune recruitment (Page 4). We have also toned down the language around preservation of tissue architecture and microenvironmental interactions to more accurately reflect the experimental context (Page 4). Importantly, we now explicitly state that, due to the absence of systemic circulation and whole-organ physiology, PDOCs have a limited capacity to recapitulate *in vivo* pharmacodynamics and pharmacokinetics. In addition, we expanded the discussion to better contextualize PDOCs within the broader landscape of available models, highlighting their complementary role alongside organoid-based systems (Page 5). This revised section emphasizes both the strengths and the limitations of PDOCs, aiming to provide a more accurate and informative overview for a broad readership.

- In "Single-cell and single-nucleus transcriptomics" section it is not mentioned what can be the caveats of snRNAseq, for instance, access to the good reference data.

We thank the reviewer for this important suggestion. We have revised the section to explicitly include key limitations of snRNA-seq. Specifically, we now highlight that the absence of cytoplasmic RNA leads to reduced transcript capture, resulting in sparser and noisier data compared with scRNA-seq. We also note that this can affect the detection of subtle cell states and the inference of genomic features such as copy-number variations, and that snRNA-seq findings do not always fully recapitulate scRNA-seq observations. Accordingly, we emphasize that results derived from snRNA-seq should be interpreted with caution. These additions are now incorporated in the revised manuscript (see updated paragraph in the "Single-cell and single-nucleus transcriptomics" section, page 6).

- It is unclear why the authors focus on spatial transcriptomics as a tool to characterize tumor microenvironment. While it is a current trend to implement these commercialized technologies, it is important to highlight publications which benchmark those and mention other, more affordable techniques, which can be informative for patient samples at levels other than transcriptome. For instance, non-antibody based spatial proteomics (LCM LC-MS, MALDI) and spatial metabolomics, lipidomics (MALDI- TOF).

We thank the reviewer for this insightful comment. In response, we have revised the manuscript to better contextualize spatial transcriptomics within the broader landscape of spatially resolved approaches. Specifically, we have expanded the section on spatial proteomics to include discussion of non-antibody-based methodologies such as laser capture microdissection coupled to LC–MS/MS and MALDI-based mass spectrometry imaging, as well as their potential applicability to brain metastasis research. We also highlight the current lack of studies applying these techniques to human BrM samples and contrast this with existing bulk proteomics efforts. These additions (see revised paragraphs in the section "Current spatial proteomics approaches", page 8) aim to provide a more comprehensive overview of available technologies, including complementary and potentially more accessible approaches beyond transcriptomics.

- The authors mention IMC and iterative staining approaches. It will be more informative to briefly compare these methods. There are critical differences, such as availability of metal-conjugated antibodies, size of scanning area, tissue damage, resolution, scanning time.

We thank the reviewer for this helpful suggestion. In response, we have revised the manuscript to include a direct comparison between IMC and iterative fluorescence-based approaches. Specifically, we now discuss key differences in resolution, scanning area, acquisition speed, antibody flexibility, and technical limitations such as autofluorescence, spectral overlap, and tissue degradation, as well as the destructive and ROI-focused nature of IMC. We have also added a brief note on the trade-offs of non-antibody-based proteomics approaches. These additions can be found in the revised section on "Technical and analytical challenges", page 9.

- Lastly, sections discussing liquid biopsies are also very descriptive, lacking details on important points. Are there any biomarkers for following brain metastases? If they can't be measured with routine methods, can we learn from other fields (eg. neurodegeneration)? Apart from the discussed biosensors, authors could mention applicability of other methods (colorimetric, fluorescence-based assays, ELISA) and developments of sensitive proteomics workflows already implemented for clinical trial samples, such as SISCAPA method (Mabrouk OS.2020 https://www.frontiersin.org/journals/neuroscience/articles/10.3389/fnins.2020.00526/f ull).

We thank the reviewer for this helpful comment. We have revised the text to further discuss the challenges and limitations associated with translating the many published biomarkers into clinical practice. In addition, we have added a new table (Table 2) highlighting several recent examples of biomarkers derived from different sources, along with their potential clinical implications if successfully translated into practice.

Minor comments:

- The review process will benefit from the line numbering to navigate the text. Done

- Sections titles lack clarity. For example, "Exploiting liquid biopsies for brain metastasis" does not specify the purpose. We have updated the text to "Towards clinical translation of liquid biopsies for brain metastases"

- In the statement in page 3 last paragraph, which combines information about rapid autopsy programmes. Are the authors sure that their statement regarding sample access is accurate for all the programmes mentioned? As, for instance, UPTIDER does not directly have access

to matched primary tumors and metastases, but it is possible to retrieve archived tumor samples from pathology department (mentioned in Guekens T. et al, 2024).

We thank the reviewer for the careful assessment of the information provided on existing rapid autopsy programs. Since detailed publications describing the structure and content of these programs are currently limited, and because these initiatives are highly active and continuously evolving, we believe it is most appropriate to provide a general overview of their existence and activities. This approach allows interested readers to identify relevant programs and directly contact the respective initiatives regarding specific questions or areas of interest.

- What are called "functional data" in page 5 top paragraph?

We assume that the reviewer refers to this sentence: “Patient-derived materials are increasingly leveraged not only as descriptive specimens but also as functional platforms to generate actionable data” This has been changed to: “Patient-derived materials are increasingly used not only as specimens for generating descriptive data (e.g. validating protein expression in human tissues), but also as functional platforms for generating actionable insights. For example, assessing the effect of an inhibitor in patient-derived organoids (PDOCs) can enable the identification of molecular signatures that stratify patients into likely responders and non-responders.

- Placement of some references needs to be adjusted. In particular, in "Emerging modelling strategies" section - in the first sentence references should be in the end of the sentence and (Krieg et al, 2025) reference should be placed prior comma. Same applies to (Wang et al., 2024) later in the text.

Thank you. This has been done.

21st May 2026

Dear Prof. Medyouf, Dear Hind,

Thank you for submitting your revised article to EMBO Molecular Medicine. I am pleased to inform you that we will be able to accept your Perspective for publication once the following editorial matters have been addressed:

1. Please remove "Unpublished data" as per our guidelines, citation of unpublished data/data not shown is not permitted
2. Please provide a short text for the legend of figure 3.
3. Please note that references are missing.

Your figures have been sent to our graphic designer to be redrawn (Figures 2 and 3), and they will be in touch with you for approval of the final figures.

Looking forward to receiving your revised manuscript at your earliest convenience.

With kind regards,

Lise

Lise Roth, Ph.D
Senior Editor
EMBO Molecular Medicine

The authors addressed the remaining editorial issues.

22nd May 2026

Dear Prof. Medyouf, Dear Hind,

Thank you for submitting your revised files. I am pleased to inform you that your manuscript has now been accepted for publication and will be transferred to our publisher once the redrawn figures have been finalized and approved by you.

Your manuscript will be processed for publication by EMBO Press. It will be copy edited and you will receive page proofs prior to publication. Please note that you will be contacted by Springer Nature Author Services to complete licensing information.
This Perspective article is free of charge.

If you have any questions, please do not hesitate to contact the Editorial Office.

Thank you for your interesting contribution to EMBO Molecular Medicine!

With kind regards,

Lise

Lise Roth, Ph.D
Senior Editor
EMBO Molecular Medicine
